# Diffusion in biological media: a comprehensive numerical-analytical study via surface analysis and diffusivities calculation

**DOI:** 10.1038/s41598-024-67348-4

**Published:** 2024-07-17

**Authors:** Juan Ignacio González Pacheco, Mariela Beatriz Maldonado

**Affiliations:** 1 Department of Chemical Engineering, Mendoza Regional Faculty, National Technological University, C. Rodriguez 273, M5502AJE Mendoza, Argentina; 2 CONICET, National Scientific and Technical Research Council, Mendoza Technological Scientific Centre, Av. Ruiz Leal S/N - Parque Gral. San Martín, M5502IRA Mendoza, Argentina

**Keywords:** Mathematical modelling, Mass transfer phenomena, Polyols, Plant-based dyes, Diffusion coefficients, Natural food production, Chemical engineering, Mathematics and computing, Applied mathematics

## Abstract

The study of diffusion in biological materials is crucial for fields like food science, engineering, and pharmaceuticals. Research that combines numerical and analytical methods is needed to better understand diffusive phenomena across various dimensions and under variable boundary conditions within food matrices. This study aims to bridge this gap by examining the diffusion of substances through biological materials analytically and numerically, calculating diffusivity and conducting surface analysis. The research proposes a process for sweetening Bing-type cherries (*Prunus avium*) using sucrose/xylitol solutions and a staining technique utilising erythrosine and red gardenia at varying concentrations (119, 238 and 357 ppm) and temperatures (40, 50 and 60 °C). Given the fruit's epidermis resistance, the effective diffusivities of skin were inferior to those in flesh. Temperature and concentration synergise in enhancing diffusion coefficients and dye penetration within the food matrix (357 ppm and 60 °C). Red gardenia displayed significant temperature-dependent variation (*p* = 0.001), whereas erythrosine dye remained stable by temperature changes (*p* > 0.05). Gardenia's effective diffusivities in cherry flesh and skin, at 357 ppm and 60 °C, 3.89E−08 and 6.61E−09 m^2^/s, respectively, significantly differed from those obtained at lower temperatures and concentrations. The results highlight the temperature-concentration impacts on mass transfer calculations for food colouring processes and preservation methodologies.

## Introduction

Throughout history, the usage of plant-based dyes has been prevalent due to their diverse range of applications and benefits to living organisms; likewise, the employment of natural-based colourants has been ubiquitous across the globe, providing a sustainable alternative to synthetic dyes^1^. The organoleptic attribute of colour is an essential factor that significantly influences food's taste perception, shelf-life, quality, and functional properties. Moreover, it plays a pivotal role in determining consumer preferences^2^. Currently, there is a rising trend towards natural additives with health benefits, resulting in a surge in demand for organic and natural products to replace artificial ingredients in the food industry^3–5^.

On the other hand, synthetic dyes are widely used in the food industry due to their versatility; however, several studies have revealed a potential correlation between their consumption and harmful effects^6^. These include allergic reactions^7^, behavioural and neurocognitive impacts^8^, hyperactivity^9^, defects in DNA^10^, as well as other chronic diseases^4^. It is essential to consider the medium and long-term health implications of synthetic dyes and consider alternative options in the food industry to ensure the safety and well-being of consumers.

Polyols, a class of sugar alcohols, are increasingly being employed as a substitute for conventional sugar in various food products owing to their low caloric content^11,12^. These compounds, comprising mannitol, xylitol, erythritol, maltitol, lactitol, and isomaltitol, occur naturally in several fruits and vegetables and are extensively incorporated in processed foods. Among the various polyols, xylitol is regarded as the sweetest and has a sweetness level equivalent to sucrose but provides three times fewer calories^13^. The use of polyols in the food industry has gained significant traction, propelled by the surging demand for low-calorie sweeteners as a substitute for traditional sugar-based products, further, given their manifold benefits and the negative connotations associated with sugary foods, polyols have revolutionised the global food market^14–16^.

Concerning the impregnation processes of fruits, namely cherries, in sweetener solutions^17–19^, it is essential to consider properly handling colourants in candied cherries due to their limited seasonality. The processing of these fruits involves placing them in solutions containing sulphur anhydride, resulting in the removal of their natural colours through a transformation into leuco bases, followed by the restoration of their pigmentation. Additionally, Giusti and Wrolstad^20,21^, as well as Sigurdson et al.^22^ conducted a comprehensive study to develop a stable, vibrant red colour for maraschino-type cherries using radish extract. This study stands as one of the few existing precedents for developing candied cherries stained with natural colourants based on available scientific data.

Numerous mathematical models have been utilised to quantify diffusion phenomena, such as Fickian^23,24^ and non-Fickian models^25^, diffusion in multicomponent mixtures^26^, numerical solution^27^, etcetera. Further, several studies on the calculation of diffusivities in foods have been developed, for instance, concomitant diffusion of salts in mushrooms^28^, calculation of diffusion coefficients during drying of chilli by microwaves and under sun^29^, the modelling of the mass diffusion phenomenon during the enrichment of food matrices by bioactive substances^30^, as well as calculation of diffusion coefficients during convective drying conditions and ultrasound-assisted osmotic dehydration of kiwi^31^ and eggplant^32^, among others. Treatment of Santa Maria pears in the 0.3% w/v gelatin solution for 30 min, caused an increase in the diffusion coefficient from 2.523E−9 m^2^/s to 3.250E−9 m^2^/s^33^. According to Fick's second law, the diffusion coefficient of microwave-blanched green almond that was exposed to the starch solution at higher microwave power (600 W) and higher drying temperature (110℃) significantly increased compared with the non-starch solution (control sample)^34^.

The ongoing study is centred on the examination of mass transfer pertaining to erythrosine and red gardenia dyes in candied cherries with the use of sucrose and xylitol. This exploration encompasses variations in concentration and temperature, with the ultimate goal of effecting a technological substitution of artificial dye with a natural alternative^35–37^. Fick's second law is employed to calculate effective diffusion coefficients^38,39^, and the concentration profiles are optimised using numerical solutions via surface plots^40^ to compare the behaviour of natural gardenia dye with erythrosine. The research addresses a significant gap in the current understanding of diffusion in biological materials and its application in food colourant processes. By examining the diffusion of substances through biological materials via diffusivity calculation and surface analysis, this study aims to contribute to the knowledge with regard to the mass transfer phenomena in natural food production. The proposed sweetening process using a combination of sucrose and xylitol, and a staining technique utilising erythrosine and red gardenia at varying concentrations and temperatures fills a gap in the existing literature. This approach will provide insights into the development of natural food colourant processes and contribute to the growing demand for organic and natural food products.

## Results

### Time-dependent study of the effect of temperature and dye concentration on the kinetics of erythrosine and red *gardenia* colourants

**Figure 1:**
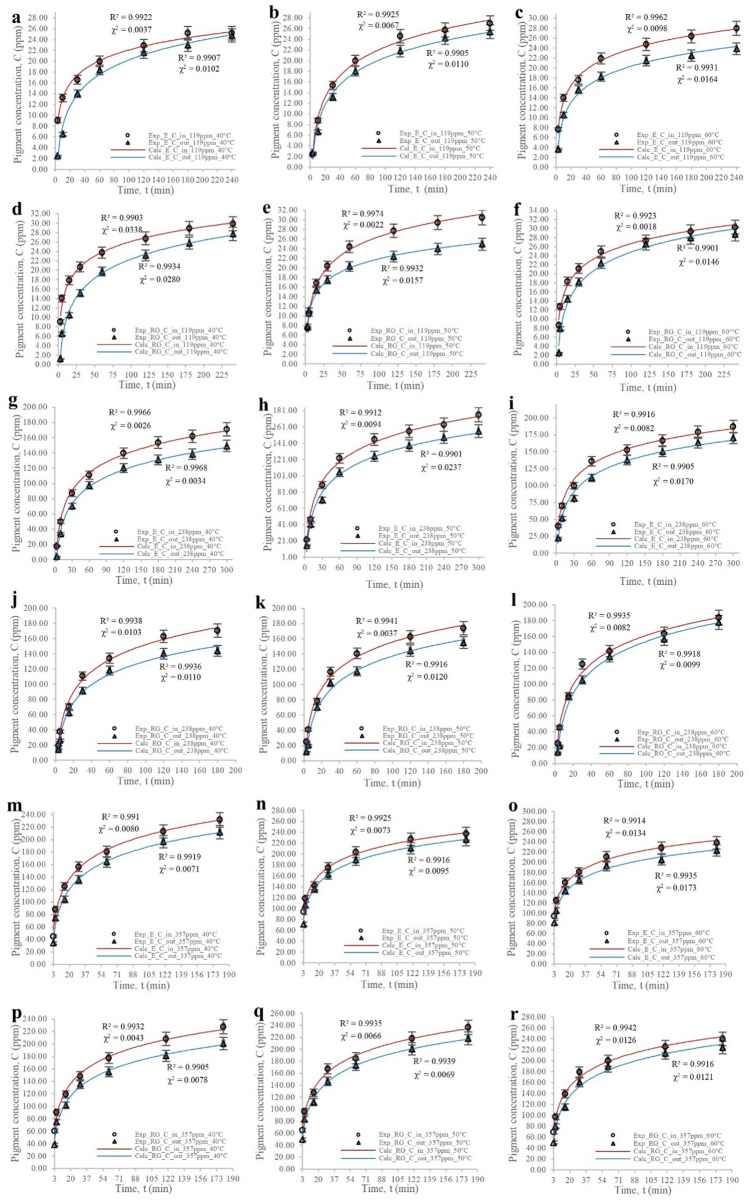
**(a-r)** Time-dependent variations in input (in) and output (out) concentrations of erythrosine (E) and red gardenia (RG) dyes at 119, 238, and 357 ppm, and temperatures of 40, 50, and 60 °C, fitted to the analytical model using 220 terms of the infinite series. The concentration values obtained through the hollow sphere model were adjusted, resulting in determination coefficients R^2^ greater than 99% and minimum chi-square values in all cases.

The ensuing study presents an analysis of the results regarding the evolution of ingress and egress concentrations of erythrosine and red gardenia dyes during the staining process within the fruit matrix, and the findings are illustrated in Fig. 1. The data presented in this figure were obtained through experimentation and analysis and provide insights into the behaviour of these dyes under different conditions of concentration and temperature.

### Diffusion coefficients and temperature-concentration influence on the transfer rates of erythrosine and red *gardenia* dyes during fruit colouring

**Figure 2:**
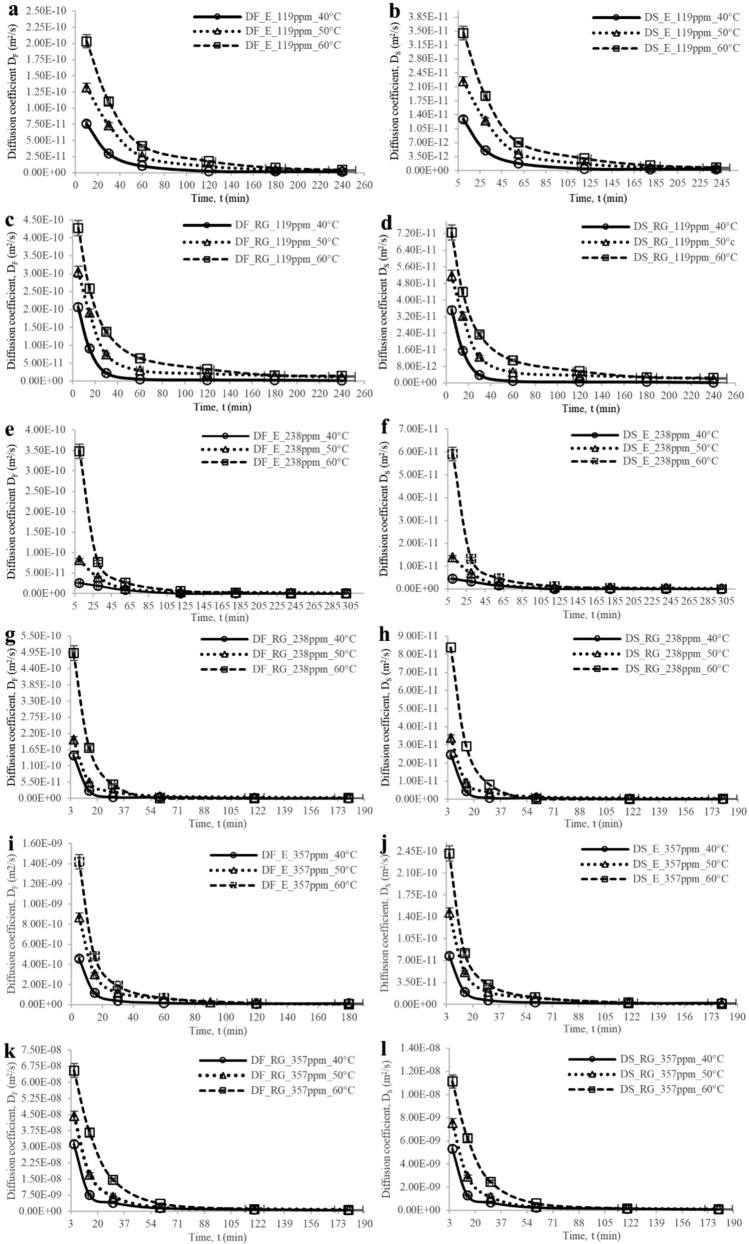
**(a-l)** Time-dependent evolution of effective diffusion coefficients for erythrosine (E) and red gardenia (RG) dyes in cherry skin (D_S_) and flesh (D_F_) at 119, 238 and 357 ppm and 40, 50, and 60 °C.

The present analysis aims to investigate the effective diffusion coefficients of erythrosine and red gardenia dyes and the influence of temperature and concentration variations on the transfer rate of species to understand the diffusion process involved through fruit colouring. The evolution of these coefficients in cherry’s flesh and skin during the colouring process is depicted in Fig. 2.

### Unveiling the space–time dynamics of dye diffusion in cherry products: a surface analysis at different temperatures and concentrations

**Figure 3:**
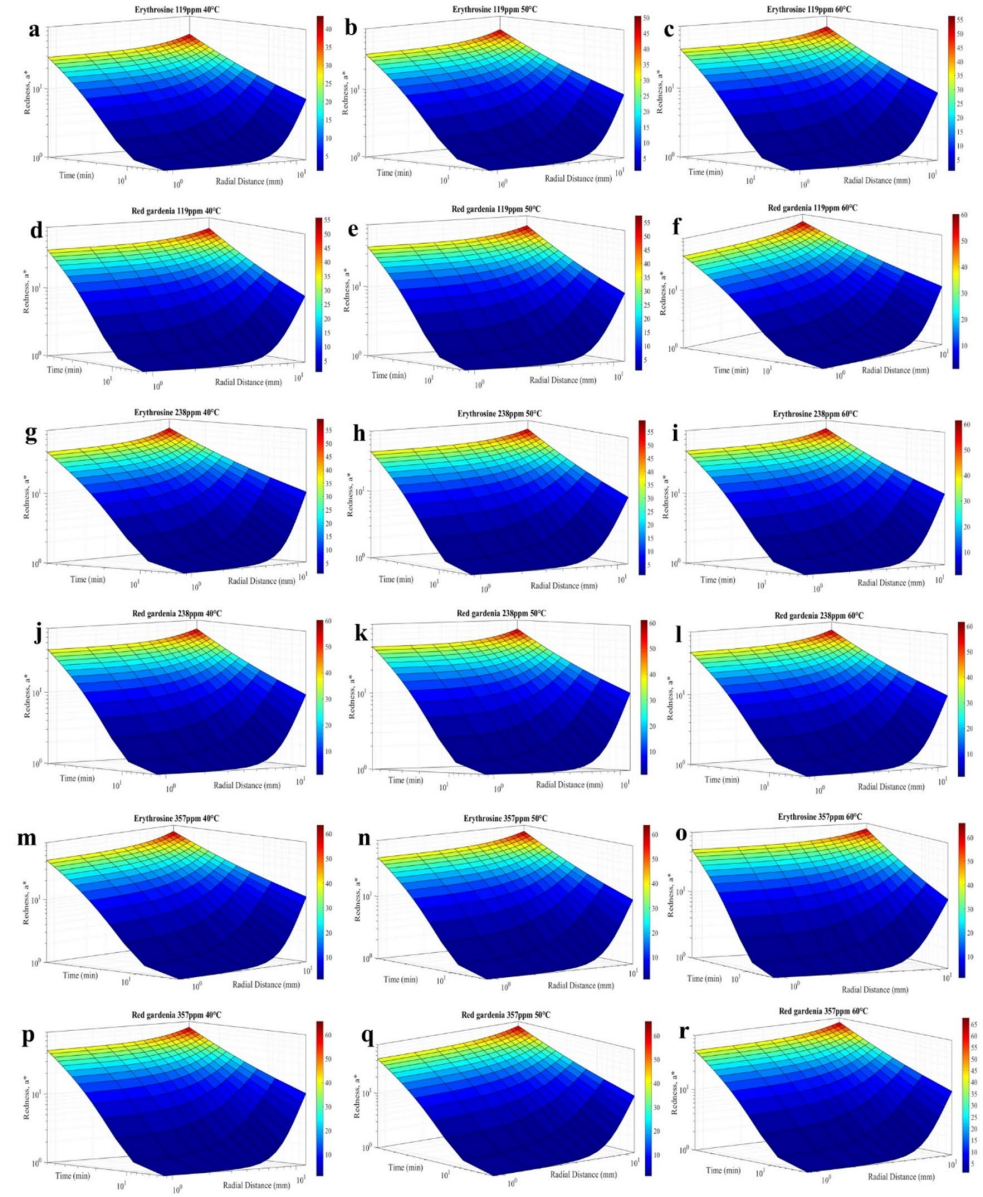
**(a-r)** Surface plots depicting the variation of erythrosine and red gardenia dyes concentration profiles, expressed as redness, as a function of time and radial distance within cherries at 40, 50, and 60 °C, with concentrations of 119, 238, and 357 ppm, respectively.

This investigation focuses on undertaking a surface analysis of the concentration profiles of erythrosine and red gardenia dyes expressed as redness, to scrutinise the effect of differing temperatures and dye concentrations on the diffusion process. The surface analysis is conducted by observing the variation of redness concerning time and radial distance within the cherries (Fig. 3).

### Statistical analysis of diffusion coefficients: influence of concentration and temperature

The subsequent information summarises the average effective diffusion coefficients of erythrosine and red gardenia dyes in cherry skin (D_S_) and flesh (D_F_) during the initial hour of the colouration process, as depicted in Table 1. Statistical parameters, namely, p-values, determination coefficients (R^2^), root mean square error (RMSE) and chi-square values (χ^2^) have been included to provide an indication of the uncertainty inherent in the mathematical model. The preliminary phase of the experiment (60 min) was chosen based on the primary molecular movement of dye species that occurred within this time frame before the system became saturated and stabilised, leading to significant deceleration in all diffusive transport (Discussion).

**Table 1:** Average effective diffusion coefficients of erythrosine and red gardenia dyes in cherry skin (D_S_) and flesh (D_F_) for the first 60 min after starting the colouring process at concentrations of 119, 238 and 357 ppm and 40, 50 and 60 °C, along with statistical parameters provided to indicate the uncertainty associated with the mathematical model.

Dye	T (°C)	C (ppm)	D_F_^†^ (m^2^/s)	*n*	*p*	*R*^*2*^	*RMSE*	*χ*^*2*^
Erythrosine	40	119	3.87E−11 ± 3.36E−11^a,‡^	5	> 0.05	0.9922	6.68E−02	3.70E−03
	50		7.69E−11 ± 5.34E−11^a^	5	> 0.05	0.9925	6.20E−02	6.70E−03
	60		1.18E−10 ± 8.12E−11^a^	5	> 0.05	0.9962	3.64E−02	9.80E−03
	40	238	3.79E−11 ± 8.59E−12^a^	5	> 0.05	0.9966	9.55E−02	2.60E−03
	50		8.40E−11 ± 3.50E−11^a^	5	> 0.05	0.9912	9.39E−02	9.40E−03
	60		1.51E−10 ± 1.73E−10^a^	5	> 0.05	0.9916	2.96E−02	8.20E−03
	40	357	2.04E−10 ± 2.21E−10^a^	4	> 0.05	0.9910	9.95E−02	8.00E−03
	50		4.29E−10 ± 3.89E−10^a^	4	> 0.05	0.9925	7.87E−02	7.30E−03
	60		6.96E−10 ± 6.43E−10^a^	4	> 0.05	0.9914	4.21E−02	1.34E−02

Dye	T (°C)	C (ppm)	D_S_^†^ (m^2^/s)	*n*	*p*	*R*^*2*^	*RMSE*	*χ*^*2*^
Erythrosine	40	119	6.57E−12 ± 5.71E−12^a^	4	> 0.05	0.9907	4.47E−02	1.02E−02
	50		1.31E−11 ± 9.08E−12^a^	4	> 0.05	0.9905	2.82E−01	1.10E−02
	60		2.01E−11 ± 1.38E−11^a^	4	> 0.05	0.9931	7.82E−02	1.64E−02
	40	238	8.04E−12 ± 1.46E−12^a^	4	> 0.05	0.9968	7.94E−01	3.40E−03
	50		1.55E−11 ± 5.96E−12^a^	4	> 0.05	0.9901	1.64E−01	2.37E−02
	60		2.56E−11 ± 2.93E−11^a^	4	> 0.05	0.9905	4.49E−02	1.70E−02
	40	357	3.46E−11 ± 3.76E−11^a^	4	> 0.05	0.9919	5.89E−02	7.10E−03
	50		7.28E−11 ± 6.61E−11^a^	4	> 0.05	0.9916	1.45E−01	9.50E−03
	60		1.18E−10 ± 1.09E−10^a^	4	> 0.05	0.9935	4.40E−02	1.73E−02

Dye	T(°C)	C(ppm)	D_F_^†^ (m^2^/s)	*n*	*p*	*R*^*2*^	*RMSE*	*χ*^*2*^
Red Gardenia	40	119	1.07E−10 ± 9.27E−11^a^	4	> 0.05	0.9903	3.31E−02	3.38E−02
	50		1.90E−10 ± 1.15E−10^a^	4	> 0.05	0.9974	1.26E−02	2.20E−03
	60		2.74E−10 ± 1.45E−10^a^	4	> 0.05	0.9923	5.62E−02	1.80E−03
	40	238	1.85E−10 ± 7.58E−11^a^	4	> 0.05	0.9938	9.57E−02	1.03E−02
	50		1.99E−10 ± 9.45E−11^a^	4	> 0.05	0.9941	8.47E−02	3.70E−03
	60		4.37E−10 ± 2.31E−10^a^	4	> 0.05	0.9935	2.82E−02	8.20E−03
	40	357	1.43E−08 ± 1.48E−08^ab^	4	0.001	0.9932	5.58E−02	4.30E−03
	50		2.28E−08 ± 1.95E−08^ab^	4	0.001	0.9935	5.32E−01	6.60E−03
	60		3.89E−08 ± 2.55E−08^b^	4	0.001	0.9942	7.32E−02	1.26E−02

Dye	T(°C)	C(ppm)	D_S_^†^ (m^2^/s)	*n*	*p*	*R*^*2*^	*RMSE*	*χ*^*2*^
Red Gardenia	40	119	1.81E−11 ± 1.57E−11^a^	5	> 0.05	0.9934	7.05E−02	2.80E−02
	50		3.23E−11 ± 3.23E−11^a^	5	> 0.05	0.9932	4.77E−02	1.57E−02
	60		4.66E−11 ± 2.47E−11^a^	5	> 0.05	0.9901	9.40E−02	1.46E−02
	40	238	1.94E−11 ± 1.29E−11^a^	4	> 0.05	0.9936	6.07E−02	1.10E−02
	50		3.56E−11 ± 1.60E−11^a^	4	> 0.05	0.9916	1.91E−01	1.20E−02
	60		4.03E−11 ± 3.90E−11^a^	4	> 0.05	0.9918	1.19E−02	9.90E−03
	40	357	2.42E−09 ± 2.52E−09^ab^	4	0.002	0.9905	1.99E−02	7.80E−03
	50		3.87E−09 ± 3.30E−09^ab^	4	0.002	0.9939	8.37E−02	6.90E−03
	60		6.61E−09 ± 4.34E−09^b^	4	0.001	0.9916	8.98E−03	1.21E−02

†Effective diffusion coefficients in flesh (D_F_) and skin (D_S_) are presented as means ± SD. ‡Different letters in the same column, indicate significant differences (p < 0.05). T: trial temperature, C: dye concentration, *p*: p-values, *n*: sample size, *R*^*2*^: determination coefficient, *RMSE*: root mean square error, *χ*^*2*^: chi-square values.

## Discussion

It can be assumed that the diffusion of both dyes exhibited enhancement upon escalation of temperature, leading to higher dye concentrations^41^. Moreover, the flow-out of colourants from the fruit followed the same pattern; however, the pH level was maintained between 4.2 and 4.8 within the fruit to induce minimal precipitation of the colourant within the matrix, resulted in lower dye outlet values than their income (Fig. 1).

Furthermore, it is deduced that no sooner had the pigment exit process initiated than the matrix was entirely saturated with sugars and dye. Consequently, the tortuous network of internal canaliculi in cherries may significantly impede the diffusion of dyes, causing a noticeable decrease in the slope of the concentration curve for both the entry and exit of pigments. Hence, this indicates that the driving force decreased progressively as the process continued.

Upon examination of the initial sample, irregularities were identified in the redness values, signifying the commencement of the pigment penetration phenomenon. The non-uniformity of initial diffusion throughout the fruit was attributed to the inherent intricacy of biological matrices, resulting lack of uniformity in dye diffusion into the fruit's interior. Consequently, only a minute quantity of dye was extracted upon reaching equilibrium, as evidenced by the minimal dye concentrations in the output curve. This observation persisted until shortly before longer staining periods, typically less than 60 to 70 min. However, in the case of extended trial durations for final samples, the redness stabilised, indicating an enhancement in colour uniformity, and the amount of dye extracted from the cherry approached an asymptote. An analysis of the kinetics of dye entry indicated complete diffusion of the dye throughout the fruit. This suggests that the pigment no longer diffuses within the sample, also signifying matrix saturation with sugars.

On the one hand, it is observed that the concentration profiles at 238 ppm (Fig. 1g–l) were analogous to those obtained in an experiment conducted at 119 ppm (Fig. 1a–f), concerning concentration variations with temperature and the curves' behaviour at the beginning and end of the trial. Additionally, the study discloses that the levels of erythrosine and red gardenia concentrations were significantly higher than in the previous research conducted at 119 ppm, attributable to the present study employing a higher concentration of colourant. Noticeably, the dye input and output concentration values were more than double those obtained at 119 ppm despite the dye concentration being twice as high as that used in the previous trial (Fig. 1a–l).

It is also revealed that the concentration profiles at 357 ppm (Fig. 1m–r) were commensurate with the results obtained in experiments conducted at 119 and 238 ppm in terms of the concentration variations with temperature and the behaviour of the curves at the start and end of the experiment. Moreover, the input and output concentrations of red gardenia and erythrosine within the matrix reached levels that were superior to those obtained at 119 and 238 ppm, attributable to the use of a greater concentration of dye. Notably, the concentration used was three times that of the trial carried out at 119 ppm (Fig. 1a–f and m–r), and yet, the concentration values that entered and were emitted were larger than triple. Therefore, it can be inferred that an increase in temperature has a significant impact when greater dye concentrations are employed in the staining process^42,43^.

On the other hand, given that colourant precipitation was compelled within the cellular tissue of the cherry, coupled with the resistance to the transfer of species offered by the cherry's skin^44^, it is deemed to assume that it is retained within the fruit. As a result, the entry diffusion of dyes should be quite accessible when there is no saturation, compared to the resistance to diffusion offered when the dye exit experiments were conducted, given that these phenomena would be coinciding. Finally, the concentration values generated by the model were duly adjusted, leading to determination coefficients R^2^ exceeding 99% in all instances and minimum chi-square values.

The following discussion is centred on investigating the effective diffusion coefficients of erythrosine and red gardenia dyes, with the objective of understanding the diffusion process involved in fruit colouring (Fig. 2). It is revealed that the driving force behind the diffusion is at its highest at the initial stages of the experiment when the concentration differential between the interior and exterior of the cherry is at its maximum. However, as the process advances, this fruit's differential concentration decreases, directing to a reduction in the driving force and a subsequent decrease in the coefficients^45^. Additionally, the accumulation of sugars and dye in the interior of the fruit results in saturation, leading to a decrease in the concentration differential of the fruit.

The results suggest that temperature and concentration play a critical role in the diffusion phenomenon^46^. The diffusion coefficients were significantly higher when the dye concentration was 238 ppm (Fig. 2e–h) compared to 119 ppm (Fig. 2a–d). The dye entry and exit curves aligned well with the obtained diffusion coefficients. The highest diffusion coefficients were observed at the outset of the trial when the most entry or exit of dye was evident. Specifically, the diffusion coefficients for erythrosine at 238 ppm in flesh (Fig. 2e) were 4.50E−11, 9.00E−11, and 3.50E−10 m^2^/s at 40, 50, and 60 °C, respectively. Meanwhile, in the skin (Fig. 2f), they were 8.60E−12, 1.50E−11, and 6.00E−11 m^2^/s at 40, 50, and 60 °C, respectively. For red gardenia in cherry flesh (Fig. 2g), the diffusion coefficients were 1.60E−10, 2.00E−10, and 4.95E−10 m^2^/s at 40, 50, and 60 °C, respectively. In cherry skin (Fig. 2h), they were 2.60E−11, 3.50E−11, and 8.50E−11 m^2^/s at 40, 50, and 60 °C, respectively.

In contrast, when the system began to saturate, and the internal tortuosity of the fruit increased, both curves stabilised after the first 60 min. The effective diffusion coefficients were found to be smaller, measuring 8.72E−12, 1.17E−11, and 2.70E−11 m^2^/s at 40, 50, and 60 °C, respectively (Fig. 2e). On the other hand, in cherry skin (Fig. 2f), the coefficients were found to be 1.48E−12, 1.99E−12, and 4.59E−12 m^2^/s at 40, 50, and 60 °C. As for red gardenia in cherry flesh (Fig. 2g), the coefficients were 7.61E−13, 1.23E−12, and 2.36E−12 m^2^/s at 40, 50, and 60 °C, whereas in skin (Fig. 2h), are calculated 4.48E−13, 7.22E−13, and 7.51E−13 m^2^/s at 40, 50, and 60 °C. It is observed that coefficients for red gardenia are significantly lower than those for erythrosine at advanced colouring times, possibly indicating that gardenia diffuses at a faster rate initially, leading to intense matrix saturation; hence, lower coefficients are obtained. On the contrary, erythrosine diffuses slower, resulting in higher coefficients under the same conditions. At these instances no significant differences were appreciated, the concentration curve was the most stable when values were the highest. The findings also revealed that the red gardenia had more substantial effective diffusion coefficients, one order of magnitude greater in both cherry skin and flesh, with the main molecular movement of species in the system occurring up to approximately 40 minutes^47^. Contrary to erythrosine, the phenomenon stabilised after about 60 min, possibly due to the smaller size of the red gardenia molecule compared to erythrosine^48^.

Furthermore, it was observed that the effective diffusion coefficients in cherry skin were one and up to two orders of magnitude lower than those obtained in cherry flesh (Fig. 2a–h). This suggests that the fruit epidermis presents a barrier effect to the diffusion of molecules^44^, as previously mentioned. The epidermis is composed of more extensive and tighter cells than that of the parenchyma, which are more closely arranged. This characteristic of the epidermis, coupled with the cuticle layer, presents a barrier effect to the diffusion of substances that differs from the diffusion in the mesocarp.

An investigation conducted by Silva et al.^49^ employed a convective-type boundary condition to model both pure diffusive transport and convective phenomena. The study underscored the presence of external resistance to mass transfer at low stirring frequencies in the extraction process of anthocyanin dye from jambolan (*Syzygium cumini* (L.)). Similarly, our current work introduces a method for computing effective diffusivities and concentration profiles in cherries (*Prunus avium*), assuming purely diffusive transport and disregarding external resistance to mass transfer. The advocated boundary condition (Eq. 5) in our study is deemed appropriate, given a large Biot (Bi) number at a high stirring frequency of 220 rpm, with the sole variable necessitating determination being the effective mass diffusivity. Notwithstanding, it is our intent to investigate convective boundary conditions in future work to elucidate the transport mechanism of substances at low stirring velocities.

Reiterating, the trial results indicate that the rate of species transfer phenomena is chiefly determined by the combination of temperature and concentration. It was observed that the diffusion coefficients were considerably superior at 357 ppm (Fig. 2i–l) than those obtained at 119 and 238 ppm dye concentrations. Additionally, it was observed that the evolution of the dye input and output curves concurred with the changes noted in the effective diffusion coefficients, further corroborating the higher coefficients at the experiment's commencement and lower coefficients at its conclusion, as previously discussed.

As stated earlier, it was noted that the effective diffusion coefficients in the skin, both in erythrosine and red gardenia, were inferior by one to two orders of magnitude to those obtained in cherry flesh because of the resistance offered by the fruit's epidermis. Additionally, the diffusion coefficients noted at 357 ppm were between three and four orders of magnitude greater than those obtained at 119 and 238 ppm, within the initial hour of commencing the colouring, ranging from 3.89E−08 to 6.61E−09 m^2^/s for red gardenia and 6.96E−10 to 1.18E−10 m^2^/s for erythrosine, in flesh and skin, respectively (Table 1). Our results are in agreement with the results reported by Kian-Pour N.^34^, which showed as the drying temperature of pretreated fresh green almonds in starch solution increased from 70℃ to 110℃, the diffusion coefficient increased from 3.502E−9 m^2^/s to 6.434E−9 m^2^/s.

The effective diffusivities in food tissues, particularly those obtained by Salehi et al.^31,50^ are comparable to the latter mentioned in the work. The values for water in apple slices flesh range from 1.48E10–10 to 4.62E10–10 m^2^/s, whilst in kiwi slices flesh, they range from 9.05E10–11 to 2.93E10–10 m^2^/s. These diffusivities are akin to those obtained with erythrosine. In an investigation conducted by Pezo et al.^51^ the osmotic dehydration process was examined across various sweet potato cultivars utilising sugar beet molasses. The study revealed effective moisture diffusivity values ranging from 1.85E − 08 to 4.83E − 08 m^2^/s. Additionally, Khoualdia et al.^52^ determined average effective diffusion coefficients for water loss and solid gain during the osmotic dehydration of Tunisian pomegranate arils in sucrose osmotic solution, yielding values of 8.30E − 09 and 4.60E − 09 m^2^/s, respectively. These findings are consistent with those observed with red gardenia.

According to the analysis, the observed diffusion coefficients in the skin and flesh are also consistent with the calculated values by da Conceição Silva et al.^53^, which span from 1.00E−12 m^2^/s to 1.00E−08 m^2^/s, respectively. Therefore, it can be concluded that concentration plays the most significant role in the diffusive transport of species, which can be optimised by temperature to generate a synergistic effect.

On the other hand, utilising surface plots^54^ as a function of the parameter a* concerning time and radial distance within the cherry at 40, 50, and 60 °C, it is depicted in Fig. 3 the concentration profiles of erythrosine and gardenia red at 119, 238 and 357 ppm (Methods). The profiles indicate that the diffusion of both dyes is progressive concerning the internal radius and time, with higher redness values appearing in the periphery. By implementing the Robin condition, Eq. (6), the double diffusion boundary could be evidenced by penetration of the dyes at the flesh-skin interface due to major redness values obtained at the cherry's skin (Fig. 3a–r). Furthermore, superior values of the redness parameter, a*, were attained at 60 °C, whilst inferior values were obtained at 40 °C in both dyes, thereby visualising the temperature-accelerating effect in the staining process within the cherry^55^.

Moreover, it can be observed that the natural dye, red gardenia, demonstrated a greater redness than erythrosine at similar impregnation times, indicating a faster diffusion rate. This observation could be attributed to the smaller size and molecular weight of the natural red gardenia dye (388.37 g/mol) compared to that of the artificial dye erythrosine (879.86 g/mol)^48^, as mentioned earlier.

The behaviour observed at 238 ppm (Fig. 3g–l) was similar to that obtained at 119 ppm (Fig. 3a–f), where the highest redness values were noticed at the flesh-skin interface, approximately 12 mm, measured from where the flesh begins. Notably, the double diffusion boundary of dye species in the skin was also verified^43^, Robin boundary condition, given that the skin resists the transfer of species while allowing the passage of a smaller flow of colouring inside the fruit, in addition to the primary flow that occurs through the flesh. The progressive increase in redness as the staining phenomenon progressed and the dye entered the matrix was also observed; therefore, the surface graphs would assertively illustrate the diffusive physical phenomenon.

Furthermore, the redness values were higher than those obtained at 119 ppm, indicating that the temperature and the concentration provide a synergistic effect by notably increasing the dye concentration, diffusion coefficients, and redness within the fruit (Fig. 3a–f and g–l). On the other hand, considering the variation profile of redness for the red gardenia dye values higher than those of the erythrosine dye were also obtained, justified by what was previously discussed.

Regarding the results obtained from the experimentation at 357 ppm (Fig. 3m–r) respecting 119 (Fig. 3a–f) and 238 ppm (Fig. 3g–l), the study findings successfully reproduced all the observed phenomena, including acquiring redness parameter profiles of erythrosine and red gardenia. As discussed earlier, the trial outcomes revealed significant insights into the mass transfer of erythrosine and red gardenia within a food matrix. Specifically, the study found that the skin of the food matrix acted as a double boundary diffusion for dye species. The highest redness values were observed at the flesh-skin interface, indicating a considerable interaction between the dyes and the food matrix. It was further identified that the diffusion coefficients for red gardenia dye were higher than those for erythrosine, suggesting an increased tendency for the former to move within the food matrix.

The optimal conditions for mass transfer of erythrosine and red gardenia within a food matrix impregnated with sucrose and xylitol were 357 ppm and 60°C^56^. These conditions enhanced all quantification parameters involved in the present study based on the concentration ranges and temperatures used.

On the other hand, a summary of the average effective diffusion coefficients of the dyes erythrosine and red gardenia in cherry skin (D_S_) and flesh (D_F_) during the initial 60-min period of the colouring process is presented in Table 1. Additionally, statistical parameters such as *p*-values, determination coefficients (*R*^*2*^), root mean square error (*RMSE*), and chi-square values (*χ*^*2*^) are provided to indicate the uncertainty associated with the mathematical model. The results indicate a good fit, with coefficients of determination above 99.5% for certain experiments, and above 99% for all cases. Chi-square values were optimised to minimise the associated uncertainty, and the obtained *RMSE* values were also minimised through the adjustment of the analytical model using the least squares and minimum chi-square methods. Furthermore, high stirring frequencies (220 rpm) led to a large Biot number case, resulting in decreased resistance to mass flow in the boundary skin-hypertonic solution^49^. Consequently, it can be inferred that the cherry skin adopts the dye concentration of the sweetening solution.

The preliminary phase of the experiment was designated based on the principal molecular movement of dye species that takes place within this time frame prior to the system reaching saturation and stability, leading to a significant deceleration in all diffusive transport.

The results indicate that temperature and concentration have a combined influence in enhancing diffusion coefficients and dye penetration within the food matrix. Even minute variations in any of these factors can impact dye mass transfer^57^. Whereas calculated diffusivities for the erythrosine dye were higher at superior temperatures and concentrations, the differences were not statistically significant (*p* > 0.05), indicating this dye's stability against temperature changes. On the other hand, significant differences (p < 0.05) were observed in the calculations obtained with red gardenia when varying the temperature.

The diffusion coefficients obtained at 357 ppm and 60 °C in cherry flesh (*p* = 0.001) and skin (*p* = 0.002) were significantly different from those obtained at 40 and 50 °C, as well as those calculated in skin and flesh of cherries coloured with erythrosine at 119, 238 and 357 ppm and 40, 50 and 60 °C. This might indicate the role of temperature variation in accelerating the colouring process with this pigment, provided that concentration values of 357 ppm or higher are used.

## Concluding remarks

An analysis of numerical and analytical solutions of the diffusion equation via surface analysis and diffusivity calculation in biological media is presented. The study revealed that the interplay of temperature and concentration influences diffusion coefficients and dye penetration within the food matrix. It can be concluded that the diffusion rate of the natural red gardenia dye is faster compared to that of the artificial dye erythrosine. The diffusion coefficients for the red gardenia dye at 357 ppm, 3.89E−08 to 6.61E−09 m^2^/s and 6.96E−10 to 3.46E−11 m^2^/s for erythrosine, in flesh and skin, respectively, were between two and three orders of magnitude greater than those for erythrosine, indicating an increased tendency for the former to move within the food matrix, possibly due to its smaller size and molecular weight. The study revealed that temperature and concentration have a combined influence in enhancing diffusivity and dye penetration within the food matrix. Even minute variations in any of these factors can impact dye mass transfer. The optimal conditions for mass transfer of both dyes within a food matrix impregnated with sucrose and xylitol were identified as 357 ppm and 60 °C.

Furthermore, the study provided insights into the process of dye diffusion, including the diffusion rate, the distance covered, and the time to diffuse, which will be pivotal in optimising the dyeing process and elevating the quality of cherry products. The research aims to improve the efficiency and quality of products coloured with such dyes by providing an understanding of the dye diffusion process and discussing the usage of plant-based dyes and synthetic dyes in the food industry. These findings bear implications for the food industry, particularly in the realm of natural food production processes and the utilisation of plant-based dyes. Comprehension of temperature and concentration's impact on diffusion coefficients presents an opportunity to enhance the efficiency and sustainability of food colouring processes, aligning with the escalating consumer demand for natural and organic products.

## Materials and methods

### Materials

**Figure 4:**
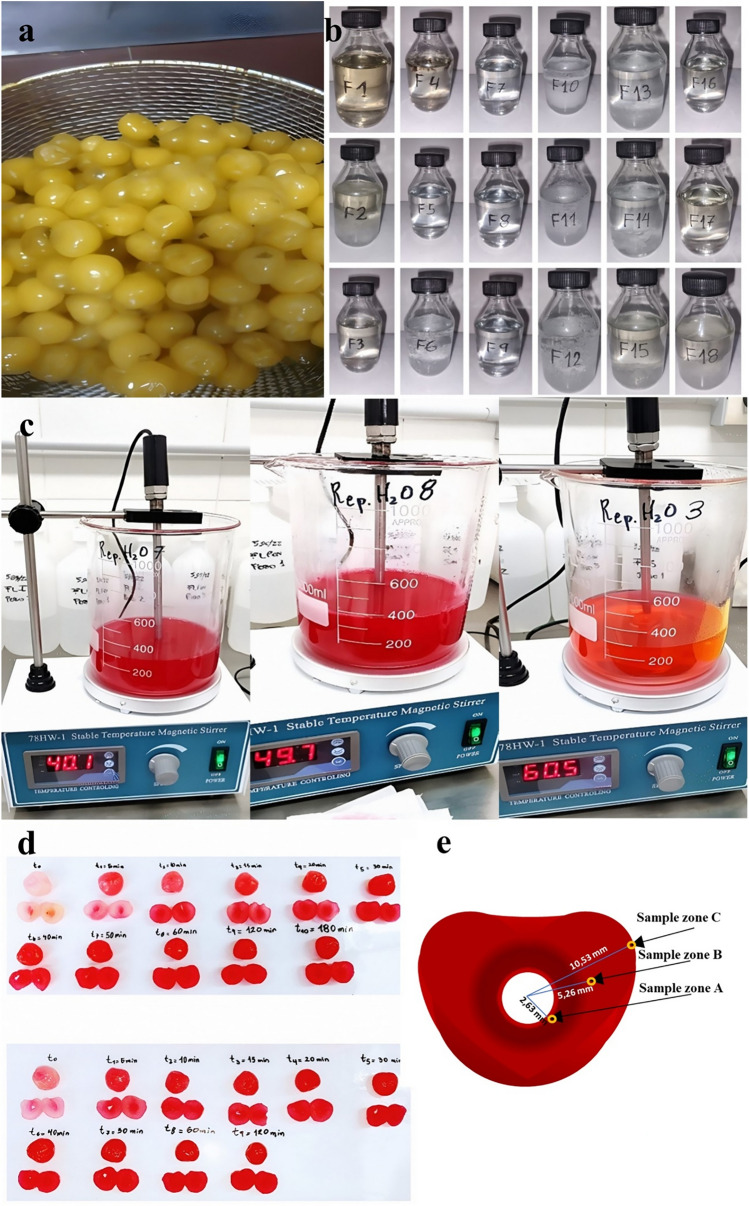
**(a-e)** Desulphited cherries after 24 h in water baths (a). Illustration of the formulations prepared constituted by sucrose and polyol solutions (b). Cherry staining trials by varying temperature, dyes, and concentration (c). Sample of cherries regularly extracted prior to measuring the L, a, and b parameters of the CIELAB colour space (d). Spacing of triplicate sampling of L, a, and b parameters of the CIELAB colour space in cherries (e).

Firstly, a batch of 10 kg of *Prunus avium* cultivar Bing was procured from Luján de Cuyo, Mayor Drummond, Mendoza, Argentina (coordinates in decimal degrees: − 33.00443364309908; − 68.86583423899584). Lactitol powder (DuPont Corporation, Wilmington, Delaware, USA), Maltitol powder (Shandong Lujian Biological Technology Co. Ltd, China), Xylitol powder (Shandong Lujian Biological Technology Co. Ltd, China) and combinations, as well as Erythrosine powder dye (Macsen Labs, N.K Agrawal Group, India) and Red Gardenia powder dye (Omya Inc., Cincinnati, Ohio, USA) were used in this study. The cherries were carefully calibrated (27 mm), pitted and desulphited (sulphur anhydride at 1500 ppm) by immersing them in tap water baths at room temperature for 24 h, with water renewal every 4 h, as illustrated in Fig. 4a.

### Methods

Fruits were plunged into sugary hypertonic solutions of low primary concentration (25 Bx). Subsequently, the concentration of the solution was systematically augmented by 10 Bx daily until it reached a final concentration of 65 Bx. This process was devised to attain the desired concentration of 55 Bx within the fruit (Slow method).

It is important to note that the staining process was executed when the concentration of the hypertonic solution reached 35 Bx. After reaching this specific concentration level, the matrix was left to rest in the solution for 24 h before proceeding with the following impregnation process. This process was repeated after each increase in the concentration of the sweetening solution until the desired concentration of 55 Bx was ultimately achieved in order to obtain candied cherries. Statement for plant material: our study complies with relevant institutional, national, and international guidelines and legislation.

Prior to initiating the impregnation process in sweetener solutions, 18 formulations were prepared. These formulations were constituted by sucrose and polyols, such as lactitol, maltitol, and xylitol, and combinations, as previously mentioned. The concentration of solutions was selected according to Maldonado et al.^58^, by dissolving the correct amount of polyols in distilled water using a stable temperature magnetic stirrer (model PIOWAY 78HW-1, BioSmartest, Norces, Argentina) at 60 °C and 220 rpm to achieve a total or partial substitution of sucrose in solution (Table 2) and (Fig. 4b). These formulations were replicated to ensure the reproducibility of the test results, thereby confirming that crystallization did not occur in the selected formulation. These were as follows:

**Table 2:** Formulations of sucrose and polyalcohol solutions prepared for candying cherries.

Formulation (form i)	Composition	Concentration (% w/V)
form 1	Sucrose	100
form 2	Lactitol	100
form 3	Maltitol	25
form 4	Maltitol	50
form 5	Maltitol	75
form 6	Maltitol	100
form 7	Xylitol	25
form 8	Xylitol	50
form 9	Xylitol	75
form 10	Xylitol	100
form 11	Maltitol/Xylitol	75/25
form 12	Maltitol/Xylitol	80/20
form 13	Maltitol/Xylitol	90/10
form 14	Maltitol/Xylitol	95/5
form 15	Lactitol/Xylitol	75/25
form 16	Lactitol/Xylitol	80/20
form 17	Lactitol/Xylitol	90/10
form 18	Lactitol/Xylitol	95/5

Where “form” 1,2,3 up to 18 was the notation employed to name the eighteen formulations of sweetening solutions.

A combination of sucrose and xylitol was the formulation utilised for candying the cherries, aiming to perform a partial replacement of table sugar^59^, thus obtaining a sweetening solution containing sucrose – xylitol 50% (w/V)^60^, since its mixture in these proportions did not generate the formation of precipitate or crystals during six months of storage at room temperature of 20 ± 2°C^61^.

#### Impregnations and staining process

The candying process was initiated through the employment of a multiple impregnation method, commonly referred to as the "French or Slow Method," as per the methodology proposed by Maldonado and González Pacheco^62^. The initial stage involves boiling and cooling a sweetener solution with a concentration of 25 Bx (first impregnation) to approximately 50 °C to prevent the occurrence of plasmolysis and the development of wrinkles in the fruit. Subsequently, the cherries were introduced into a 35 Bx syrup (second impregnation) under identical conditions to those of the preceding day. During this procedure, erythrosine and red gardenia were added at 119, 238 and 357 ppm to stain the fruits and observe their influence on concentration. The solution is maintained at a steady temperature of 40, 50, and 60 °C and subjected to constant agitation through a thermostatic magnetic heating stirrer PIOWAY 78 HW-1 at 220 rpm (Fig. 4c), ensuring the solution’s homogeneity.

To regulate the pH of the sweetening solution, the integration of 0.9 mL of 10% (w/V) citric acid or 0.3 mL of 10% (w/V) NaHCO_3_ was implemented^63^. This measure facilitates the maintenance of a pH range between 4.2 and 4.8, which induces slight precipitation of the pigment within the cellular tissue of the cherry and enhances the fruit's vivid and visually appealing hues^64^.

#### Sampling

Two cherry samples were randomly selected from multiple container zones, as depicted in Fig. 4d. These samples' cross-sections were acquired with a scalpel's assistance, and the parameters *L*, a*, and b* of the colour space were subsequently measured^65^. The luminosity (*L*), redness or greenness (a*), and blueness or yellowness (b*) (referred to in combination as CIELAB) were measured at three specific points, namely 2.63 mm, 5.26 mm, and 10.53 mm on average, calculated from the centre of the cherry, using a Konica Minolta CR-400 colourimeter and an illuminant D65 (natural light). These measurements were taken from where the flesh begins, as illustrated in Fig. 4e. Additionally, a whole cherry was used to measure the same parameters on the outside to discern the colouring progress during the diffusion of the pigments.

**Figure 5:**
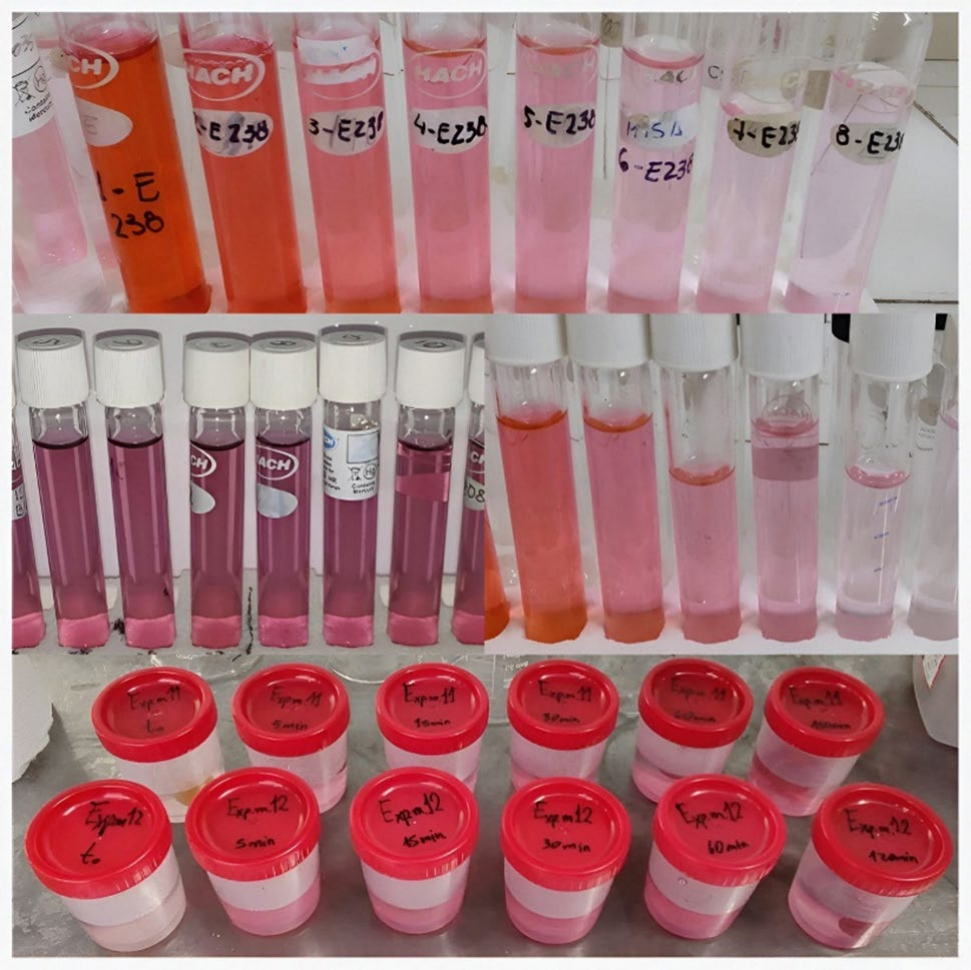
Cherry samples collected to measure dye output by UV–VIS spectrophotometry.

Furthermore, a mathematical proposition was later formulated concerning these dye species' diffusion phenomenon with a double diffusion boundary. Therefore, it was imperative to extract cherry samples and preserve them in the same original hypertonic solution (without dye) to determine the output of these species across concentration gradients (Fig. 5). By introducing 0.9 ml of 10% (w/V) citric acid or 0.3 ml of 10% (w/V) NaHCO_3_ to the cherry, as previously mentioned, a pH range of 4.2 to 4.8 was achieved, halting dye release as the dye precipitated within the cellular tissue of the fruit. This method allows for the containment of dye within the fruit's matrix during the colouring stage, thus facilitating the kinetics of dye entry. Manipulating the pH within this specific range represents a significant technological advancement, particularly in preventing cocktail cherries from staining neighbouring fruits with colouration.

**Figure 6:**
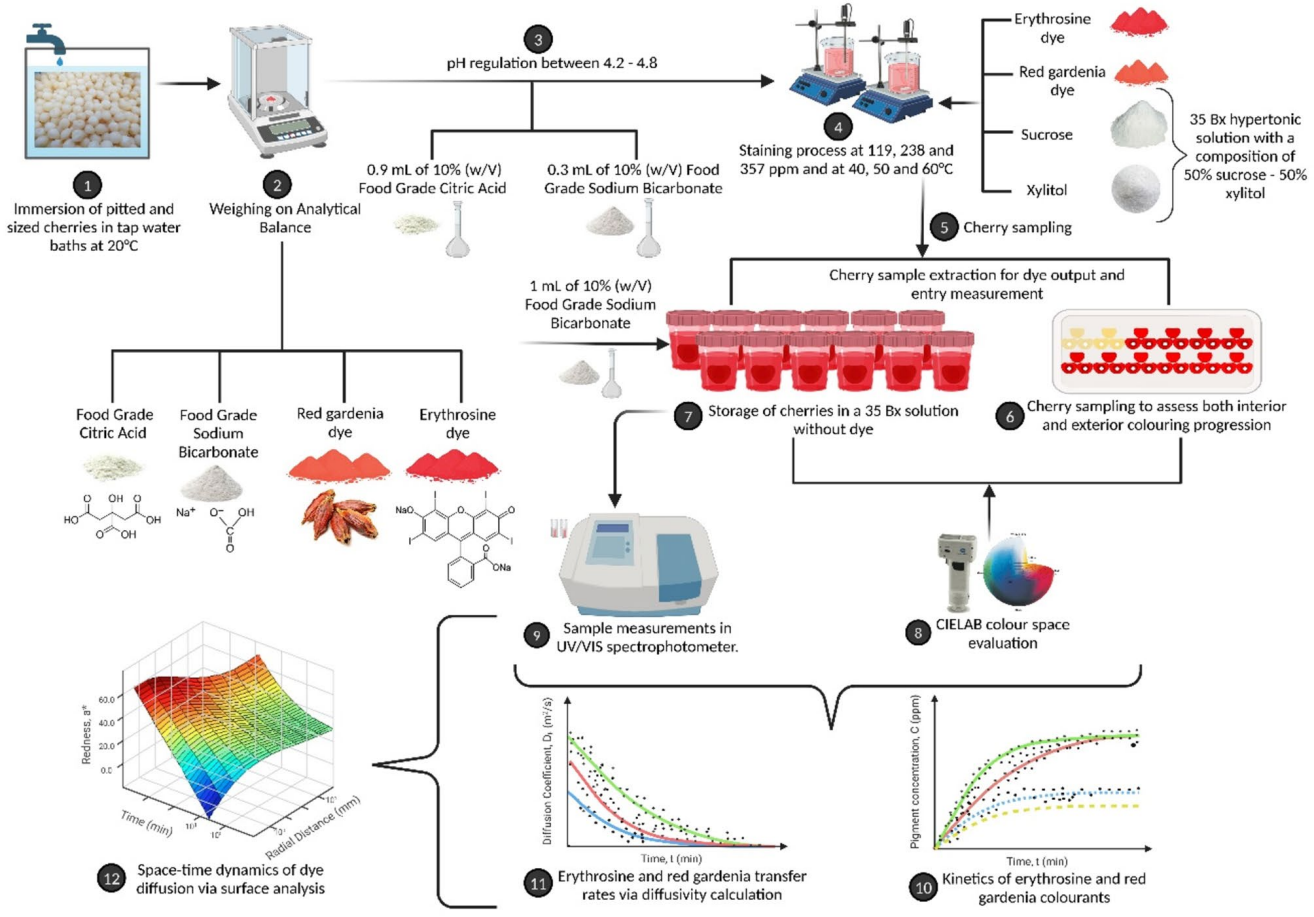
Schematic diagram delineating the process of cherry colouration, encompassing the following steps: sample collection, colour analysis, UV/VIS spectrophotometry, determination of effective diffusivities, dye input and output kinetics, as well as the generation of surface graphs and concentration profiles within the fruit. Created with BioRender.com (publishing license: IJ26X22SH1).

Conversely, to determine the kinetics of dye output, 1 ml of 10% (w/V) NaHCO3 was added to raise the pH between 5.5 and 6. This was conducted in the original hypertonic solution of 35 Bx of 50% xylitol—50% sucrose without dye, thereby recreating the initial conditions prior to the colouring process. Under these conditions, the equilibrium of dye concentrations inside the cherry and the sweetening solution was achieved after approximately 24 h at room temperature of 20 ± 2 °C (Fig. 6).

In the initial stages of the experiment, it was determined that the initial sample size for assessing the cherries should exceed triplicate due to the variability in external cherry colour (n = 5). This decision stemmed from the inherent complexity of biological matrices, highlighting the non-uniform diffusion of dyes within the intricate tissue matrix. However, as the experiment progressed and the cherries underwent dyeing, there was a noticeable enhancement in colour uniformity, reflected in closely clustered redness values and the resultant vivid red pigmentation of the fruit (Fig. 4d). Consequently, a decision was made to reduce the sample size to three measurements (n = 3).

The solutions were measured in a Perkin Elmer Lambda 35 UV–VIS spectrophotometer at 530 nm^66^, and the measurements were carried out in triplicate. Moreover, an analysis was conducted to examine the impact of concentration and temperature on the ingress and egress kinetics of dyes within the fruit during the candying process. The assessment also encompassed an inquiry into the effective diffusivity of the dye within the fruit's skin and flesh. Additionally, numerical resolutions were determined to visually represent the penetration phenomena and associated concentration profiles at varying temperatures and presence of dye during the progressive advancement of the dye diffusion process within the biological medium, as depicted in the schematic diagram featured in Fig. 6.

#### Mathematical modelling

The experimental data underwent mathematical analysis during the colouring and impregnation or osmotic dehydration process in sweetened solutions. This process involved the adjustment to a differential equation, which governs the molecular diffusion through the cherry matrix regarded as a solid porous hollow sphere.

##### Theory

Considering the transfer of species, the equation of the Reynolds transport theorem was utilised. Assuming the total molar concentration of the binary mixture constant and independent diffusion coefficients to spatial coordinates, the equation can be simplified and expressed tensorly, Eq. (1), as follows^67^.

1 $$\frac{{\partial C_{i} }}{\partial t} + \left( {\overline{v}^{*} .\nabla C_{i} } \right) = {\mathcal{D}}_{ij} \nabla^{2} C_{i} + R_{i} - \frac{{C_{i} }}{C}\left( {R_{i} + R_{j} } \right)$$

where $C_{i}$ is the molar concentration of species *i* in the binary mixture, ${\mathcal{D}}_{ij}$ is the effective diffusion coefficient for a binary system, $\overline{v}^{*}$ is the local average molar velocity of the mixture, the production rate of the species *i* or *j* per unit volume due to chemical reactions are denoted by $R_{i}$ or $R_{j}$, and *t* is time.

Thus, the dimensionless, one-dimensional diffusion equation, with constant effective coefficients in the flesh (*D*_*F*_) and skin (*D*_*S*_), considering only the molecular diffusion of dyes as the transport mechanism within the fruit, while disregarding the generation term of substances through chemical reactions, can be regarded as a partial differential equation, Eq. (2), as follows^68–70^:

2 $$\frac{{\partial {\mathcal{C}}}}{\partial \Theta } = \frac{2}{R}\frac{{\partial {\mathcal{C}}}}{\partial R} + \frac{{\partial^{2} {\mathcal{C}}}}{{\partial R^{2} }}$$

where $$\Theta = \frac{{{\mathcal{D}}_{ij} t}}{{r_{o}^{2} }}$$*;* $$R = \frac{r}{{r_{0} }}$$*;* $${\mathcal{C}} = \frac{{C_{\left( t \right)} - C_{s} }}{{C_{i} - C_{s} }}$$.

Note that theta, $\Theta$, is a non-dimensional time, $R$ is a non-dimensional radius, ${\mathcal{C}}$ is the non-dimensional concentration, $C_{\left( t \right)}$ is the dye concentration within the cherry’s flesh as time progressed, $C_{s}$ the dye concentration in the cherry's skin in contact with the sweetening solution^71^, and $C_{i}$ the dye concentration within the cherry’s flesh, at the beginning of the experiment.

**Figure 7:**
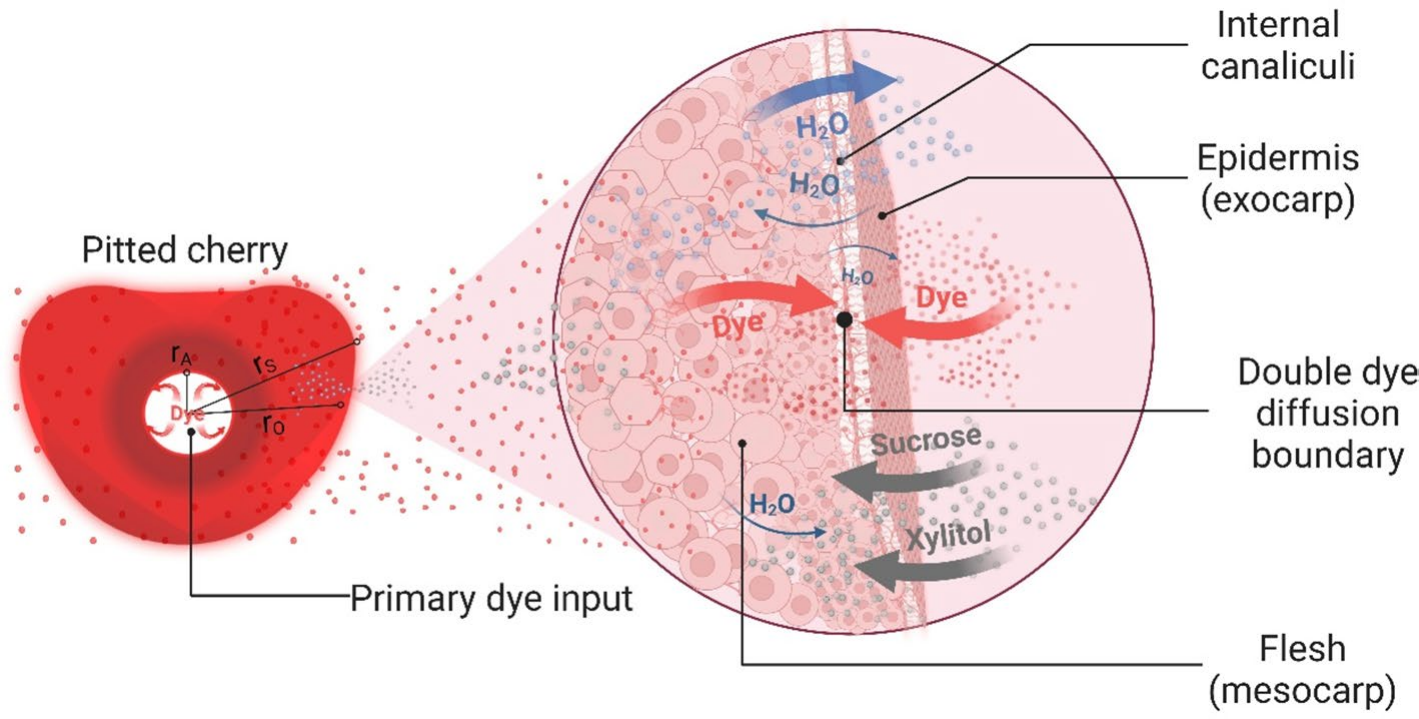
Schematic representation of the internal radii of the cherry and an enlargement of the epidermis zone and its surrounding area, presenting the formation of a double diffusion boundary. The visualisation illustrates a simple model of the dye input flowing through the flesh and the cherry skin. Created with BioRender.com (publishing license: KB26TRPPY7).

It was deemed appropriate to assume that the accumulation of solutes in the skin, represented by $r_{S} - r_{O}$, and accordingly the resistance to species transfer, was insignificant in comparison to the flux through the porous surface and the flux itself, given by the cherry skin thickness of 0.0004 m. The cherries’ flesh was considered homogeneous and isotropic, and the diffusion process exhibited radial symmetry with unidirectional diffusion. Due to the great agitation of the sweetener solution surrounding the internal and external surfaces, the concentration in these areas immediately acquires the concentration of the liquid. Furthermore, the epidermis’s thickness was much smaller than the radii of the inner and outer faces. Thus, the difference in surface areas could be disregarded, and the thickness assumed as $r_{S} - r_{O}$ (Fig. 7). It should be noted that despite creating 18 distinct sweetening formulations, the cherries are exclusively immersed in the hypertonic sucrose-xylitol solution for the candying process.

##### Calculation

An alternative version of Eq. (2) can be obtained by fitting the redness parameter, a*, using the least squares method with a straight-line approach, compared to the current erythrosine or red gardenia dye concentration, Eq. (3), thereby establishing a correlation that enabled the experimental data to be fitted to the general equation governing species transport.

3 $$\frac{{\partial a^{*} }}{\partial \Theta } = \frac{2}{R}\frac{{\partial a^{*} }}{\partial R} + \frac{{\partial^{2} a^{*} }}{{\partial R^{2} }}$$

Subjected to the following initial and boundary conditions:

4 $$I.C.: At \Theta = 0\quad a^{*} = \frac{{a_{i}^{*} - a_{s}^{*} }}{{a_{i}^{*} - a_{s}^{*} }} = 1\quad at\;A \le R \le 1$$

5 $$B. C. 1: At \Theta > 0\quad a^{*} = \frac{{a_{s}^{*} - a_{s}^{*} }}{{a_{i}^{*} - a_{s}^{*} }} = 0\quad at\;R = A$$

6 $$B.C. 2:At \Theta > 0 \quad \frac{{\partial a^{*} }}{\partial R} + \frac{{D_{S } }}{{D_{F} }}\left( {\frac{{r_{0} }}{{r_{s} - r_{0} }}} \right)a^{*} = 0\quad at\quad R = 1$$

where $$\Theta = \frac{{{\mathcal{D}}_{ij} t}}{{r_{o}^{2} }}$$*;* $$R = \frac{r}{{r_{0} }}$$*;* $$A = \frac{{r_{A} }}{{r_{0} }}$$*;*$$a^{*} = \frac{{a_{\left( t \right)}^{*} - a_{s}^{*} }}{{a_{i}^{*} - a_{s}^{*} }}$$.

Note that $A$, is a non-dimensional radius, given by the relation between $r_{A}$ and $r_{0}$, $a_{\left( t \right)}^{*}$ is the redness within the cherry’s flesh as time progressed, $a_{s}^{*}$ the redness in the cherry's skin in contact with the sweetening solution, and $a_{i}^{*}$ the redness within the cherry’s flesh, at the beginning of the experiment.

Then, an infinite series can be expressed, Eq. (7), representing the evolution of the dimensionless volumetric parameter ${\text{a}}_{{{\text{V}}\left( \Theta \right)}}^{*}$ in relation to dimensionless time Θ, modified to that presented by Maldonado and González Pacheco^48^.

7 $${\text{a}}_{{{\text{V}}\left( \Theta \right)}}^{*} = \frac{3}{{\left( {1 - A^{ 3} } \right)}}\mathop \sum \limits_{1}^{\infty } \left\{ {\frac{1}{b}\left[ {\mathop \int \limits_{A}^{1} R^{2}\upphi _{n\left( R \right)} dR} \right]^{2} - \frac{{WA_{0} }}{{\lambda_{n}^{2} - A_{1} }}\left[ {e^{{(\lambda_{n}^{2} - A_{1} )\Theta }} - 1} \right]\mathop \int \limits_{A}^{1} R^{2}\upphi _{n\left( R \right)} dR} \right\}e^{{ - \lambda_{n}^{2} \Theta }}$$

To determine the effective diffusion coefficients and eigenvalues $\lambda_{n}$, an iterative nonlinear regression method was employed using the least squares method in conjunction with *GraphPad PRISM *^*©*^* Software (V9.5.1, MA, USA)*. For a large mass transfer Biot number (given conditions mentioned earlier) corresponding to the specified boundary condition, Eq. (5), considerable truncation errors may arise, particularly during the initial stages of the process. Nonlinear regression relying on a limited number of terms in the series of analytical solutions for determining effective diffusivity is deemed unsuitable for achieving precise results. Consequently, the first 220 terms of the analytical solution of the diffusion equation for spherical geometry were incorporated alongside the optimiser^49,72^.

Regarding further details of the mathematical description, see Supplementary Information.

#### Statistical analysis

In light of the unequal sample sizes, a Tukey–Kramer HSD multiple comparisons test, along with the Scheffe test, was implemented. Additionally, we applied the minimum chi-square method to determine diffusivity and model fits^49,72^. Statistical significance was determined by a *p*-value less than 0.05 in the ANOVA analysis, using *IBM*^*Ⓡ*^* SPSS*^*Ⓡ*^* Statistics* software (*V22.0, IBM*^*Ⓡ*^* Co., NY, USA*) to determine significant differences in the mean of the coefficients. The results are presented as mean ± standard deviation (SD).

#### Numerical solution

Aiming to predict the concentration profiles and visualise the gradual entrance of both dyes, the *MATLAB® R2017a* software (*V9.2, The MathWorks Inc., MA, USA*) was employed. The latter was configured using the *MATLAB® PDE Solver*, based on the assumption of spherical coordinates with azimuthal and zenith angular symmetries to adjust the expression, Eq. (3), to Dirichlet boundary conditions, Eqs. (4) and (5), and Robin condition or mixed boundary condition, Eq. (6), by fitting a hollow sphere model featuring a double diffusion boundary at the cherry flesh-skin interface. The problem was solved numerically using the finite element method. A mesh with 15 equally spaced points was utilised in the spatial interval [0,12]. The time interval was [0,60], considering the primary movement that occurs during the initial stages of the process (Discussion).

The solver encodes the coefficients for PDEs (partial differential equations) of the form, Eq. (8):

8 $$c\left( {x,t,u,\frac{\partial u}{{\partial x}}} \right)\frac{\partial u}{{\partial t}} = x^{ - m} \frac{\partial }{\partial x}\left( {x^{m} f\left( {x,t,u,\frac{\partial u}{{\partial x}}} \right)} \right) + s\left( {x,t,u,\frac{\partial u}{{\partial x}}} \right)$$

The terms included in the equation are:

$$f\left( {x,t,u,\frac{\partial u}{{\partial x}}} \right)\;{\text{is}}\;{\text{a}}\,{\text{flux}}\,{\text{term}}.$$

$$s\left( {x,t,u,\frac{\partial u}{{\partial x}}} \right)\;{\text{is}}\;{\text{a}}\;{\text{source}}\;{\text{term}}.$$

The variable m is utilised to indicate the three possible symmetry types for an object, namely slab, cylindrical, or spherical symmetry. These classifications are assigned the values of 0, 1, or 2, respectively. In the current context, it has been established that the value of m is equal to 2.

The coupling of partial derivatives concerning time is subject to the restriction of being multiplicatively tied to a diagonal matrix $c\left( {x,t,u,\frac{\partial u}{{\partial x}}} \right)$, whose diagonal entries are either zero or positive. A diagonal entry of zero indicates an elliptic equation, whereas all other entries correspond to parabolic equations, with at least one parabolic equation being mandatory. Furthermore, a diagonal entry of *c* corresponding to a parabolic equation can vanish at isolated *x* values, provided that those *x* values are mesh points.

The components of the solution, as articulated in Eq. (8), are in accordance with boundary conditions of the form, as specified in Eq. (9):

9 $$p\left( {x,t,u} \right) + q\left( {x,t} \right)f\left( {x,t,u,\frac{\partial u}{{\partial x}}} \right) = 0$$

The matrix $q\left( {x,t} \right)$ comprises diagonal elements that are either zero or always non-zero. It is noteworthy that the boundary conditions are expressed in terms of the flux *f*, rather than the partial derivative of u with respect to *x*. Additionally, among the two coefficients $p\left( {x,t,u} \right)$ and $q\left( {x,t} \right)$, only *p* is dependent on *u*.

### Statement for plant material

The study complies with relevant institutional, national, and international guidelines and legislation.

### Supplementary Information

Supplementary Information.

## Data Availability

The dataset pertaining to impregnations and staining at different concentrations and temperatures, along with coding in MATLAB® PDE Solver, is available at http://hdl.handle.net/11336/232171.
