# Supplementary material for: Diffusion in biological media: a comprehensive numerical-analytical study via surface analysis and diffusivities calculation
Source: Sci Rep. 2024 Jul 17;14:16513. doi: 10.1038/s41598-024-67348-4 (PMC11255277; doi:10.1038/s41598-024-67348-4)
Supplement: Supplementary file 1 — Supplementary Information. [file 41598_2024_67348_MOESM1_ESM.docx]

**Supplementary Information**

***Appendix A***

The mathematical equations presented herewith are an integral part of the description that models the mass transfer phenomenon, considering the first 220 terms of the infinite series.

$$W=\frac{1}{b}\sqrt{\frac{2}{\pi\lambda_{n}}}\left\{ \frac{\mathcal{D}_{S}}{\mathcal{D}_{F}}\left( \frac{r_{0}}{r_{s}-r_{0}} \right)\left[ \sin(\lambda_{n})+\left( \frac{\lambda_{n}\cos\left( \lambda_{n} \right)-\sin\left( \lambda_{n} \right)}{\lambda_{n}\sin\left( \lambda_{n} \right)+\cos\left( \lambda_{n} \right)} \right)\cos\left( \lambda_{n} \right) \right] \right\} (A.1)$$

$\int_{A}^{1} R^{2}ɸ_{n\left( R \right)}dR=\sqrt{\frac{2}{\pi\lambda_{n}}}\left\{ \frac{1}{{\lambda_{n}}^{2}}\left[ \sin\left( \lambda_{n} \right)-\sin\left( \lambda_{n}A \right) \right]-\frac{1}{\lambda_{n}}\left[ \cos\left( \lambda_{n} \right)-A\cos\left( \lambda_{n}A \right) \right]+\frac{\left[ \lambda_{n}\cos\left( \lambda_{n} \right)-\sin\left( \lambda_{n} \right) \right]}{\left[ \lambda_{n}\sin\left( \lambda_{n} \right)+\cos\left( \lambda_{n} \right) \right]}\left[ \frac{1}{{\lambda_{n}}^{2}}\left[ \cos\left( \lambda_{n} \right)-\cos\left( \lambda_{n}A \right) \right]-\frac{1}{\lambda_{n}}\left[ \sin\left( \lambda_{n} \right)-A\sin\left( \lambda_{n}A \right) \right] \right] \right\} (A.2)$

$b=\frac{1}{\pi\lambda_{n}}\left\{ \left[ \left( 1-A \right)+\frac{\sin\left( 2\lambda_{n}A \right)-\sin\left( 2\lambda_{n} \right)}{2\lambda_{n}} \right]+\frac{2}{\lambda_{n}}\frac{\left[ \lambda_{n}\cos\left( \lambda_{n} \right)-\sin\left( \lambda_{n} \right) \right]}{\left[ \lambda_{n}\sin\left( \lambda_{n} \right)+\cos\left( \lambda_{n} \right) \right]}\left[ \sin^{2}\left( \lambda_{n} \right)-\sin^{2}\left( \lambda_{n}A \right) \right]+\frac{\left[ \lambda_{n}\cos\left( \lambda_{n} \right)-\sin\left( \lambda_{n} \right) \right]^{2}}{\left[ \lambda_{n}\sin\left( \lambda_{n} \right)+\cos\left( \lambda_{n} \right) \right]^{2}}\left[ \left( 1-A \right)-\frac{\sin\left( 2\lambda_{n}A \right)-\sin\left( 2\lambda_{n} \right)}{2\lambda_{n}} \right] \right\} (A.3)$
$A_{0}=\frac{6}{\left( 1-A^{3} \right)}{R_{\lambda_{n}}}^{2} (A.4)$

$A_{1}={\lambda_{n}}^{2.0031} (A.5)$

wherein

$R_{\lambda_{n}}={(B}_{3}C_{3})-\left( D_{3}E_{3} \right)+A\lambda_{1}\left( B_{3}E_{3}+D_{3}C_{3} \right) (A.6)$

In addition:

$B_{3}=\frac{\cos\left( \lambda_{n}A \right)}{\lambda_{n}} (A.7)$

$C_{3}=\frac{1}{\lambda_{n}}\left[ \sin\left( \lambda_{n} \right)-\sin\left( \lambda_{n}A \right) \right]+\left[ A\cos\left( \lambda_{n}A \right)-\cos\left( \lambda_{n} \right) \right] (A.8)$

$D_{3}=\frac{\sin\left( \lambda_{n}A \right)}{\lambda_{n}} (A.9)$

$E_{3}=\frac{1}{\lambda_{n}}\left[ \cos\left( \lambda_{n} \right)-\cos\left( \lambda_{n}A \right) \right]+\left[ \sin\left( \lambda_{n} \right)-A\sin\left( \lambda_{n}A \right) \right] (A.10)$
